# Multimodal MRI Image Decision Fusion-Based Network for Glioma Classification

**DOI:** 10.3389/fonc.2022.819673

**Published:** 2022-02-24

**Authors:** Shunchao Guo, Lihui Wang, Qijian Chen, Li Wang, Jian Zhang, Yuemin Zhu

**Affiliations:** ^1^Key Laboratory of Intelligent Medical Image Analysis and Precise Diagnosis of Guizhou Province, College of Computer Science and Technology, Guizhou University, Guiyang, China; ^2^College of Computer and Information, Qiannan Normal University for Nationalities, Duyun, China; ^3^CREATIS, CNRS UMR 5220, Inserm U1044, INSA Lyon, University of Lyon, Lyon, France

**Keywords:** glioma classification, multimodal MRI images, decision fusion, tumor segmentation, deep learning

## Abstract

**Purpose:**

Glioma is the most common primary brain tumor, with varying degrees of aggressiveness and prognosis. Accurate glioma classification is very important for treatment planning and prognosis prediction. The main purpose of this study is to design a novel effective algorithm for further improving the performance of glioma subtype classification using multimodal MRI images.

**Method:**

MRI images of four modalities for 221 glioma patients were collected from Computational Precision Medicine: Radiology-Pathology 2020 challenge, including T1, T2, T1ce, and fluid-attenuated inversion recovery (FLAIR) MRI images, to classify astrocytoma, oligodendroglioma, and glioblastoma. We proposed a multimodal MRI image decision fusion-based network for improving the glioma classification accuracy. First, the MRI images of each modality were input into a pre-trained tumor segmentation model to delineate the regions of tumor lesions. Then, the whole tumor regions were centrally clipped from original MRI images followed by max–min normalization. Subsequently, a deep learning-based network was designed based on a unified DenseNet structure, which extracts features through a series of dense blocks. After that, two fully connected layers were used to map the features into three glioma subtypes. During the training stage, we used the images of each modality after tumor segmentation to train the network to obtain its best accuracy on our testing set. During the inferring stage, a linear weighted module based on a decision fusion strategy was applied to assemble the predicted probabilities of the pre-trained models obtained in the training stage. Finally, the performance of our method was evaluated in terms of accuracy, area under the curve (AUC), sensitivity, specificity, positive predictive value (PPV), negative predictive value (NPV), etc.

**Results:**

The proposed method achieved an accuracy of 0.878, an AUC of 0.902, a sensitivity of 0.772, a specificity of 0.930, a PPV of 0.862, an NPV of 0.949, and a Cohen’s Kappa of 0.773, which showed a significantly higher performance than existing state-of-the-art methods.

**Conclusion:**

Compared with current studies, this study demonstrated the effectiveness and superiority in the overall performance of our proposed multimodal MRI image decision fusion-based network method for glioma subtype classification, which would be of enormous potential value in clinical practice.

## Introduction

Glioma is the most common primary tumor of the brain and spine, representing 80% of malignant brain tumors (1) and having varying degrees of aggressiveness and prognosis. The clinical manifestations of glioma include increased intracranial pressure, neurological/cognitive dysfunction, and seizures. According to the 2016 WHO classification of tumors of the central nervous system (CNS), glioma can be classified into astrocytoma (grade II or III), oligodendroglioma or mesenchymal oligodendroglioma (grade II or III), and glioblastoma (grade IV), depending on the pathology and molecular alterations (2). Low-grade glioma is well-differentiated and presents an aggressive growth pattern in terms of biological characteristics, whereas high-grade glioma is a malignant brain tumor that is difficult to identify and has a poor prognosis (3).

Precise glioma classification or grading is crucial for deciding the right therapeutic strategies that may further impact the prognosis process of patients (4, 5). In clinical practice, MRI is the standard medical imaging technique for brain tumor diagnosis for its advantages of relative safety and non-invasiveness as compared to pathological biopsy examinations (6). With respect to unimodal MRI images, multimodal MRI images can provide more morphological, functional, and tumor metabolic status information due to their correlation and complementary information for all types of brain tumors. Clinically, the low contrast between tumor masses and surrounding tissues as well as the varying levels of physicians’ experience may lead to misdiagnosis; more importantly, diagnosing based on manual analysis is a time-consuming procedure (7). With the development of artificial intelligence and computing facilities, computer-aided diagnosis (CAD) technology based on computer vision has been applied to many medical fields and provides help for physicians in visualization and tumor identification to improve the subjective diagnosis manually (8).

So far, the methods for brain tumor classification in the latest studies can be loosely classified into two categories: traditional machine learning methods and deep learning methods (9). Among the latest traditional machine learning methods (5, 10–12), the most commonly used one is radiomics. Radiomics uses data characterization algorithms to extract quantitative features from MRI images (13, 14), and these features usually contain complex patterns that are difficult to recognize or quantify by human eyes, such as tumor heterogeneity, infiltration, and metastasis (15). The other general method is deep learning, which was successfully applied to tumor segmentation (16), tumor classification (7, 8, 17), survival prediction (4, 18), and molecular genetic prediction (19, 20) for its powerful feature representation in medical imaging fields. Compared with radiomics-based methods, deep learning-based methods do not need domain-specific knowledge for feature extraction and outcompete the formers when experimental data are sufficient. Furthermore, considering the powerful feature learning capability of deep learning and the powerful classification capability of traditional machine learning, researchers have combined them together for glioma classification or grading (21–23).

The aim of this study was to diagnose the glioma subtype preoperatively using MRI images only for assisting in making appropriate treatment decisions. Misdiagnosis caused by inaccurate glioma prediction algorithm may lead to severe injury or death, so prediction accuracy is the most concerned performance undoubtedly. Since Computational Precision Medicine- Radiology-Pathology (CPM-RadPath) on Brain Tumor Classification challenge held in 2018, many studies have been conducted in glioma subtypes prediction using multimodal MRI images based on tumor segmentation. Pei et al. (4) proposed a 3D convolutional neural network (CNN) model for glioma classification based on tumor segmentation results from the CANet model, and experimental results demonstrated the effectiveness of using MRI images only. Xue et al. (24) trained a 3D residual convolutional network with MRI images for glioma classification, and the results showed that using tumor segmentation regions would get higher accuracy. Pei et al. (25) applied a 3D CNN model with MRI images for brain tumor segmentation, which distinguished brain tumors from healthy tissues, and then the segmented tumors were used for tumor subtype classification with another 3D CNN model. Yin et al. (26) achieved the first rank in CPM-RadPath 2020 using both MRI and pathological images. For multimodal MRI images, they used the pre-trained model on the Brain Tumor Segmentation (BraTS) challenge 2019 for tumor segmentation and then built a densely connected convolutional network (DenseNet) model for glioma prediction in their scheme. Although promising in their results, they concatenated the multiple modalities as different input channels such that a deep learning network could automatically learn to extract the multimodal features. With such an image fusion strategy, it is difficult to adjust the contributions of each modality for prediction results and consequently not easy to get the best classification accuracy.

Using multimodal MRI images for glioma subtype classification has great clinical potentiality and guidance value. In order to further improve the glioma subtypes prediction accuracy in clinical applications, we propose a Multimodal MRI Image Decision Fusion-based Network (MMIDFNet) based on the deep learning method. Inspired by image fusion (27), the proposed method uses a linear weighed module to assemble the models trained with a single modality together for mining their complementary predictive capabilities. To evaluate the effectiveness of our proposed method, we compared the classification performance between our method and recent state-of-the-art methods. Additionally, since radiomics-based methods are also commonly used in recent brain tumor classification studies, we also implemented a radiomics-based method as the benchmark for performance comparison with our MMIDFNet.

## Materials and Methods

### Study Cohort

Our experimental data were obtained from the CPM-RadPath challenge 2020 dataset^1^, which is supported by Medical Image Computing and Computer-Assisted Intervention Society. The dataset classified patients into three subtypes based on the WHO-CNS pathomorphological classification criteria, named Glioblastoma (abbreviated as “G”), Astrocytoma (abbreviated as “A”), and Oligodendroglioma (abbreviated as “O”) separately (25, 26). Each patient contained preoperative 3D MRI images and pathological whole slide images. MRI images comprise four different modalities of T1-weighted (T1), T2-weighted (T2), post-contrast T1-weighted (T1ce), and fluid-attenuated inversion recovery (FLAIR). Considering the purposes of our study, we just used MRI images to predict pathological subtypes. According to the dataset description, MRI images were obtained from multi-parametric MRI scans in routine clinics with 1T to 3T MRI scanners in multi-center institutions and stored in NIfTI format. All four MRI modalities were preprocessed with bias field correction, skull stripping, and co-registration into the same anatomical structure template (24). The volume size of each MRI modality data is 240 × 240 × 155, where 155 indicates the number of slices. The cohort in our experiments consisted of 221 patients collected from the original dataset, in which there were 133, 54, and 34 samples provided for subtype “G”, “A”, and “O”, respectively. To overcome the bias caused by a particular selection for the pair of training and testing sets, a 3-fold cross-validation strategy was used in this work. Specifically, the dataset was split into 3 smaller sets, the model was trained using 2 of the folds as training data, and then the trained model was validated on the remaining part of the data. The performance reported by 3-fold cross-validation was measured with the averaged evaluation indices.

### MMIDFNet Architecture

**Figure 1:**
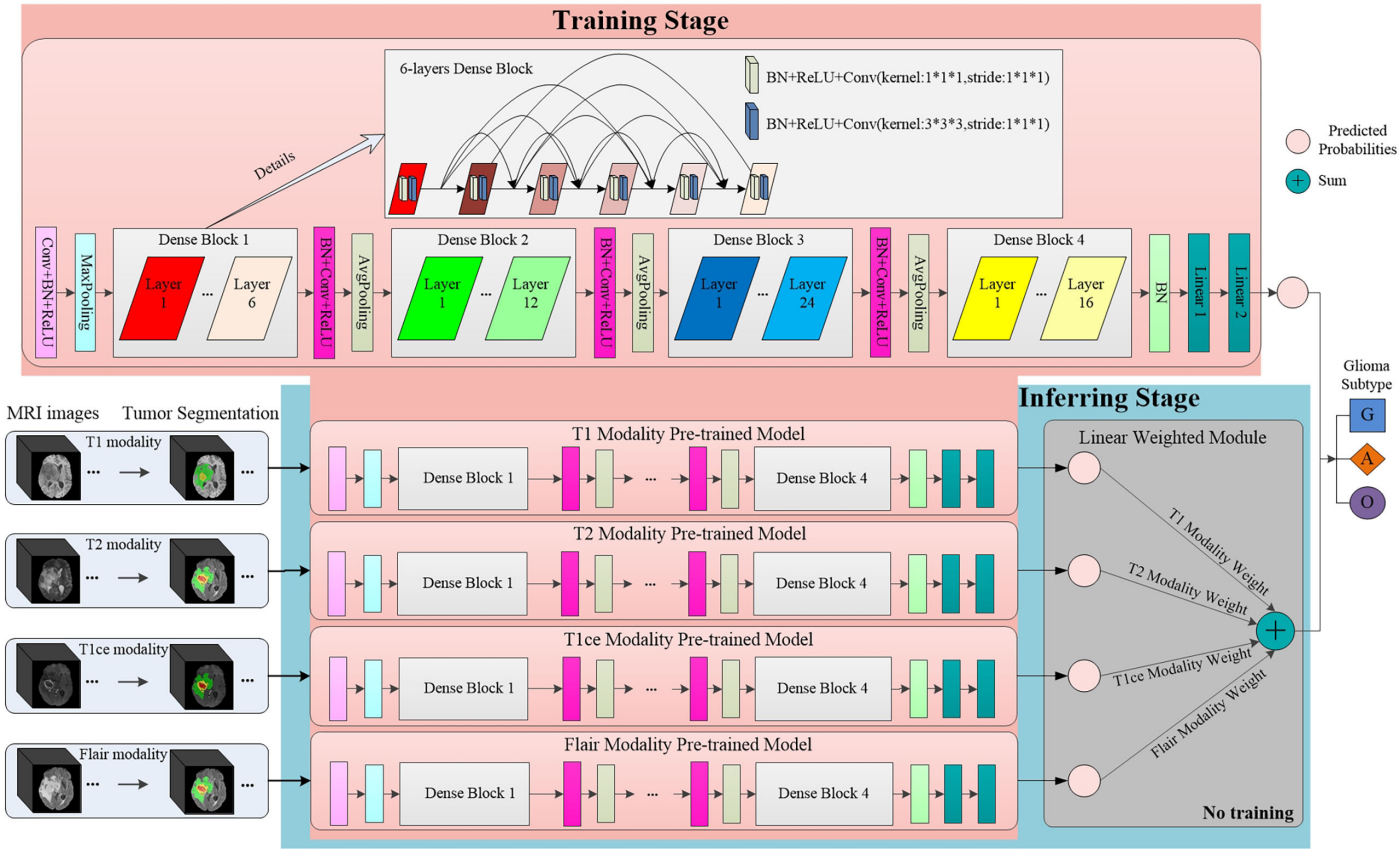
The structure of our proposed MMIDFNet.

To improve the accuracy of glioma diagnosis using multimodal MRI images, we designed the MMIDFNet for glioma subtype classification, as shown in **Figure 1**. The MMIDFNet architecture includes two parts: one is the tumor segmentation module using a pre-trained model, and the other is the two stages of unimodal model training and multimodal image decision fusion inferring module. In the training stage, we used the images of each single modality to train the network to obtain its best accuracy on the testing set. In the present work, we used images from four MRI modalities. Thus, we have four pre-trained models. In inferring stage, a decision fusion strategy was used; in other words, a linear weighted module was applied to assemble the predicted probabilities of the above four pre-trained models for each modality. Adopting the decision fusion strategy, we can fully take advantage of the complementary capabilities among unimodal models trained from different modalities. Note that the weights for the four MRI modalities in our linear weighted module did not participate in training in inferring stage.

### Tumor Segmentation

**Figure 2:**
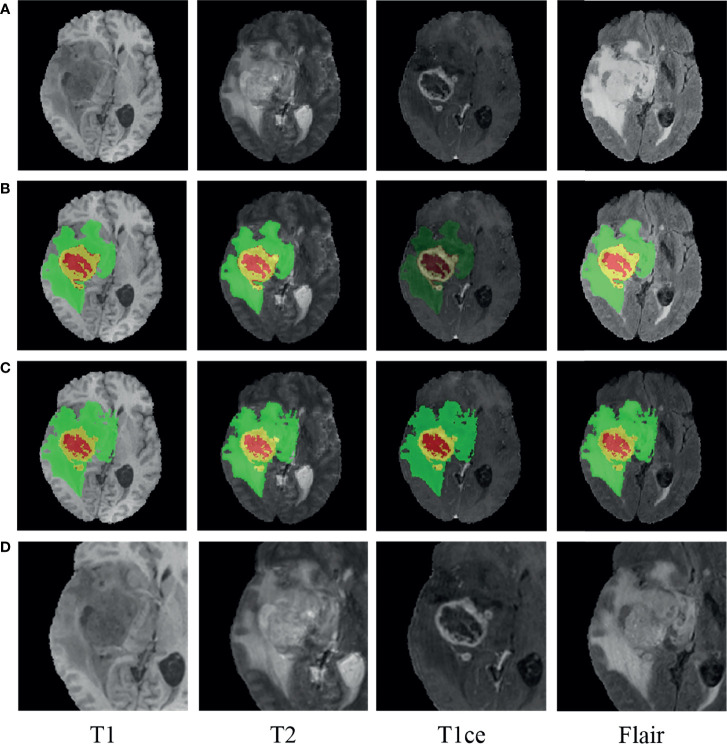
An example of glioma patients on multimodal MRI images (patient ID: CPM19_CBICA_AAB_1, Glioblastoma). **(A)** Original images. **(B)** Tumor segmentation on panel **(A)**. **(C)** Ground truth on panel **(A)**. **(D)** Normalized followed centrally clipped from panel **(A)** based on panel **(B)**.

Accurate segmentation of brain tumors from MRI images is of enormous potential value for improved diagnosis (28). It can be done automatically to cope with the time-consuming disadvantage of manual segmentation (29, 30). Considering that the MRI images in our study were also used in the BraTS challenge and that the ground truth of tumor segmentation for patients in our cohort are not all available, we used the pre-trained model on the BraTS challenge 2019 to delineate the regions of tumor lesions, which achieved the accuracy of 90.45% on the validation set (31). In the BraTS 2019 dataset, all the samples in the training set are provided with four ground truth labels for 4 regions: background (label 0), necrotic and non-enhanced tumor (label 1), peritumoral edema (label 2), and enhanced tumor (label 4). We reassigned the non-zero labels into three combined subregions, representing enhanced tumor (ET: label 4), tumor core (TC: label 1 + label 4), and whole tumor (WT: label 1 + label 2 + label 4). The WT, TC, and ET regions of the MRI images were obtained by the pre-trained segmentation model. Since glioma grows within the substance of the brain and often mixes with normal brain tissues, the surrounding area is also valuable for the assessment. Hence, we used the whole tumor regions as the segmentation regions of interest (ROIs) and centrally cropped the original image to 128 × 128 × 80. In order to make the intensities of the cropped images more homogeneous, max–min normalization was applied. Besides, for all the patients having ground truth, after carefully comparing the pairwise central locations of WT regions obtained by our tumor segmentation and the ground truth, we found that they were all consistent or nearby, which also demonstrates that our tumor segmentation scheme is feasible. A glioblastoma patient case before and after segmentation with the pre-trained model is displayed in **Figure 2** using the ITK-SNAP software. The red region indicates the necrotic and non-enhanced tumor, the yellow region the enhanced tumor, and the green region the peritumoral edema.

### Unimodal Prediction Model Building

Through stacking multiple convolutional layers together, deep neural networks (DNNs) can automatically learn discriminative features from imaging data. Among different DNN models as well as their variants, DenseNet has shown superior classification performance as it strengthens feature propagation from one dense block to the next and overcomes the problem of vanishing gradient (32). In the training stage of our MMIDFNet method, we designed a network based on unified DenseNet architecture with 121 layers (32), which included four dense blocks, and each dense block was composed of several convolutional layers (BN+ReLU+Conv). The number of convolutional layers in four dense blocks was set as 6, 12, 24, and 16. The features of images were extracted through these dense blocks. After that, two fully connected layers were used to map the features into three glioma subtypes. In order to maintain the 3D structural features of MRI images, we used them as the model input directly without converting them into 2D slices. In the training stage, the model loss used focal loss for handling class imbalance, the initial learning rate was set to 5 × 10^−4^ with the updating strategy of scheduler optimization, the optimizer used Adam algorithm with impulse, the batch size was set to 8, and the number of training epoch was set to 100. To overcome overfitting in the training process, many strategies (e.g., sample normalization, data augmentation, applying L2 normalization to model loss, designing dropout layer for the model, and setting weight decay for optimizer) were employed. Our unimodal prediction models were implemented by the PyTorch framework (version 1.4.0, Facebook), and the details of network architecture can be found in **Figure 1**. All the data augmentation strategies of dimension resizing, random rotation, random scaling, random Gaussian noise adding, and random contrast adjusting were implemented using the Medical Open Network for AI (MONAI) toolkit^2^. Since our model with a large number of parameters would lead to high computational cost, we used NVIDIA Tesla A100 GPU to reduce the running time of model training and validation.

### Multimodal Prediction Model Building

Due to the correlation among different modality images, multimodal MRI can provide help to extract features from different views and bring complementary information (33). For exploring richer patterns among multimodal MRI images to handle the issue of insufficient classification ability and generalization ability of the unimodal model, we used multimodal MRI images to build prediction models based on image decision fusion strategy. In our MMIDFNet method, since the classification capacity of different pre-trained models using a single MRI modality in the training stage is usually different and complementary, we assembled a multimodal prediction model with multimodal MRI images by calculating the linear weighted sum of predicted probabilities from the four pre-trained unimodal models trained in the training stage. Based on the heuristic searching strategy, the weights of each pre-trained unimodal model were assigned according to their classification ability. The final predicted probabilities of our MMIDFNet model for gliomas subtype classification was calculated as follows:


(1) $$fusion\_prob=\sum_{i=1}^{m}w_{i}*prob_{i}$$

where *m* is the number of pre-trained models, and *w_i_* and *prob_i_* are the weight and the prediction probability of the *i*th pre-trained model, respectively.

### Radiomics-Based Prediction Models Building

The radiomics-based method mainly includes four stages: tumor segmentation, radiomics feature extraction, feature selection, and classification model building (34). The major challenge of the radiomics-based method is how to extract features from 3D MRI images. By using the Pyradiomics^3^ package, for each original 3D MRI image, a total of 106 radiomics features were extracted based on the mask obtained in the above tumor segmentation stage. These features were composed of 18 first-order statistical features, 14 shape features, and 74 wavelet texture features. Besides, 12 other types of images (8 wavelets, gradient, mean square, root mean square, and exponential) transformed from each 3D MRI image were also used to extract radiomics features. Finally, the features of the original image as well as its 12 transformed images were concatenated together in an end-to-end manner, thus forming a total of 1,378 (106 × 13) features for each MRI image. Similarly, as for multimodal MRI images of each patient, their features were also concatenated together and formed the joint features for training a multimodal glioma prediction model. In the present work, we used the images from four MRI modalities. Hence, the dimension of the joint features is 5,512 (1,378 × 4).

Considering that redundant and irrelevant features in high-dimensional features usually influence learning accuracy (35, 36), the least absolute shrinkage and selection operator (Lasso) regression algorithm was performed to reduce feature dimension by retaining high discriminative features (5, 27). Based on the selected features, the random forest (RF) classification model, which is frequently used in the field of supervised machine learning (5, 11, 12, 30, 37), was built for our task of glioma diagnosis. The Lasso and RF algorithms were implemented using the scikit-learn library (version 0.23.1).

### Performance Metrics

The performance of our multi-class predictive models was assessed according to the commonly used accuracy, sensitivity, specificity, positive predictive value (PPV), negative predictive value (NPV), and the area under the curve (AUC) of the receiver operating characteristic (ROC). The goal of the CPM-RadPath challenge is to assess automated brain tumor classification algorithms using three metrics, namely, F1_score, Cohen’s Kappa (Kappa), and balanced accuracy (Balanced_Acc), which are sensitive to the imbalanced distribution of sample classes. Among the above metrics, F1_score and Balanced_Acc are defined as accuracy and sensitivity in multi-class metrics, respectively. The formulas for calculating the performance metrics of accuracy, sensitivity, specificity, PPV, NPV, and Kappa are given by Equations 2–7, respectively:


(2) $$Accuracy=\frac{\sum_{i=1}^{n}TP_{i}}{Num}$$

(3) $$Sensitivity=\frac{\sum_{i=1}^{n}TP_{i}/(TP_{i}+FN_{i})}{n}$$

(4) $$Specificity=\frac{\sum_{i=1}^{n}TN_{i}/(TN_{i}+FP_{i})}{n}$$

(5) $$PPV=\frac{\sum_{i=1}^{n}TP_{i}/(TP_{i}+FP_{i})}{n}$$

(6) $$NPV=\frac{\sum_{i=1}^{n}TN_{i}/(TN_{i}+FN_{i})}{n}$$

(7) $$Kappa=\frac{p_{0}-p_{e}}{1-p_{e}}$$

In Equations 2–6, *Num* is the number of samples, *TP* the true positives, *TN* the true negatives, *FP* the false positives, *FN* the false negatives, and *n* the number of sample categories. In Equation 7, *p*_0_ denotes the sum of the number of samples for each correct classification divided by the total number of samples, and *p_e_* the expected agreement when both annotators assign labels randomly (6). According to the accuracy metric, the best classifier was chosen as our predictive model for the task of glioma subtype classification.

### Statistical Analysis

Age being the only available clinical factor in the CPM-RadPath challenge dataset (6), we converted it from days to years for simplicity before analysis. The differences in age and glioma subtypes between the training and testing sets were assessed using the Mann–Whitney rank-sum test. The statistical quantifications of the performance metrics were demonstrated with 95% CI, when applicable. All statistical analyses were carried out with the Scipy module (version 1.3.1), and p-value <0.05 indicated a significant difference.

## Results

Among these retrospective patients (age ranges 17 to 85 years), the mean ± SD of age was approximately 53.8 ± 14.8. In each fold, the number of subtypes “G”, “A”, and “O” was about 60.2%, 24.4%, and 15.4%, respectively. From the p-value results of the Mann–Whitney rank-sum test, we found that there was no significant difference in pathological subtypes (0.439, 0.423, and 0.48) among each fold.

### Unimodal Prediction Models

**Figure 3:**
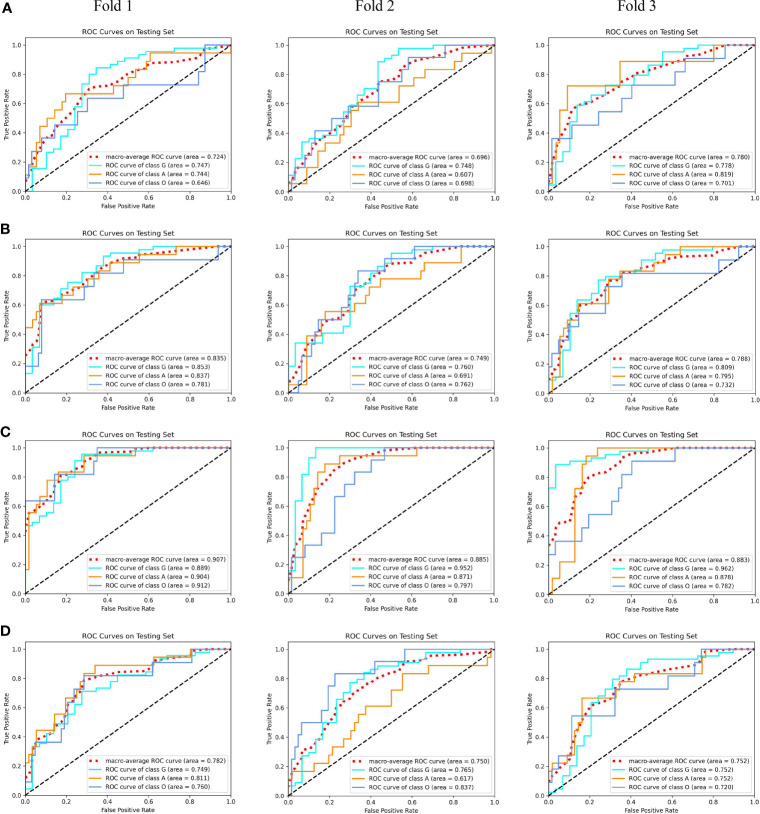
The receiver operating characteristic (ROC) curves of unimodal prediction models on three validation folds using our MMIDFNet method. **(A)** T1 modality. **(B)** T2 modality. **(C)** T1ce modality. **(D)** Flair modality.

After carefully tuning the parameters of unimodal models with our MMIDFNet method, we obtained the best pre-trained prediction models for each single modality in the training stage in turn. The ROC curves of each pre-trained unimodal model for each fold are plotted in **Figure 3**. We found that the prediction performance for each modality among different validation folds was not significantly different, which validates that the dataset selection has no significant influence on the prediction performance of our method.

In addition, the performance of unimodal prediction models using radiomics and our proposed MMIDFNet method on three validation folds is summarized in **Table 1**. We noticed that the split of the training and validation sets indeed influenced the prediction performance of both radiomics and our models, but not significantly. In general, our proposed MMIDFNet method generated better results than the radiomics method, with the highest averaged evaluation indices, except for the modalities of T1 and T2, in which the AUC, or sensitivity, or PPV was a little lower than that of radiomics. Moreover, we observed that with either our MMIDFNet method or radiomics method, using different unimodal MRI images achieved different classification performances. As for the radiomics method, among the unimodal prediction models on each fold, using the T1ce modality achieved the best-averaged accuracy of 0.815. The averaged AUC, sensitivity, specificity, PPV, and NPV were 0.868, 0.704, 0.882, 0.796, and 0.912, respectively. Meanwhile, for our MMIDFNet method, using the T1ce MRI modality achieved the best-averaged accuracy of 0.833. The averaged AUC, sensitivity, specificity, PPV, and NPV were 0.892, 0.708, 0.894, 0.817, and 0.924, respectively. This demonstrates that T1ce images may be beneficial to the glioma subtype classification.

**Table 1:** Three-fold cross-validation performance of unimodal prediction models using radiomics and our proposed MMIDFNet.

Methods	Modality	Fold	ACC	AUC	SEN	SPE	PPV	NPV
Radiomics	**T1**	1	0.730	0.734	0.546	0.792	0.807	0.865
		2	0.689	0.755	0.522	0.765	0.748	0.844
		3	0.699	0.737	0.610	0.792	0.682	0.812
		**Average**	**0.706**	**0.742**	**0.559**	**0.783**	**0.746**	**0.840**
		95% CI	[0.672, 0.740]	[0.724, 0.760]	[0.487, 0.632]	[0.758, 0.808]	[0.646, 0.846]	[0.798, 0.883]
	**T2**	1	0.703	0.775	0.554	0.787	0.657	0.824
		2	0.743	0.827	0.596	0.816	0.722	0.875
		3	0.712	0.712	0.560	0.820	0.615	0.843
		**Average**	**0.719**	**0.771**	**0.570**	**0.808**	**0.665**	**0.847**
		95% CI	[0.686, 0.753]	[0.679, 0.863]	[0.534, 0.606]	[0.779, 0.836]	[0.578, 0.751]	[0.806, 0.889]
	**T1ce**	1	0.838	0.908	0.706	0.890	0.867	0.928
		2	0.784	0.841	0.650	0.854	0.770	0.905
		3	0.822	0.856	0.756	0.903	0.752	0.902
		**Average**	**0.815**	**0.868**	**0.704**	**0.882**	**0.796**	**0.912**
		95% CI	[0.770, 0.859]	[0.812, 0.925]	[0.619, 0.789]	[0.842, 0.923]	[0.697, 0.895]	[0.889, 0.934]
	**Flair**	1	0.730	0.788	0.570	0.792	0.763	0.881
		2	0.685	0.718	0.557	0.787	0.625	0.813
		3	0.743	0.740	0.585	0.810	0.756	0.888
		**Average**	**0.719**	**0.749**	**0.571**	**0.796**	**0.715**	**0.861**
		95% CI	[0.671, 0.768]	[0.691, 0.806]	[0.548, 0.593]	[0.777, 0.816]	[0.590, 0.839]	[0.794, 0.927]
MMIDFNet	**T1**	1	0.757	0.724	0.572	0.821	0.813	0.894
		2	0.689	0.696	0.509	0.767	0.663	0.890
		3	0.712	0.780	0.516	0.777	0.701	0.873
		**Average**	**0.719**	**0.733**	**0.532**	**0.788**	**0.726**	**0.886**
		95% CI	[0.664, 0.775]	[0.665, 0.802]	[0.477, 0.588]	[0.742, 0.834]	[0.601, 0.850]	[0.868, 0.904]
	**T2**	1	0.743	0.835	0.542	0.794	0.742	0.907
		2	0.730	0.749	0.560	0.820	0.687	0.854
		3	0.726	0.788	0.591	0.822	0.642	0.853
		**Average**	**0.733**	**0.791**	**0.564**	**0.812**	**0.690**	**0.871**
		95% CI	[0.719, 0.747]	[0.722, 0.860]	[0.525, 0.604]	[0.787, 0.837]	[0.610, 0.770]	[0.822, 0.921]
	**T1ce**	1	0.838	0.907	0.764	0.885	0.842	0.909
		2	0.824	0.885	0.667	0.897	0.749	0.934
		3	0.836	0.883	0.694	0.900	0.859	0.929
		**Average**	**0.833**	**0.892**	**0.708**	**0.894**	**0.817**	**0.924**
		95% CI	[0.821, 0.845]	[0.870, 0.913]	[0.628, 0.788]	[0.881, 0.907]	[0.722, 0.911]	[0.903, 0.945]
	**Flair**	1	0.770	0.782	0.669	0.855	0.755	0.866
		2	0.703	0.750	0.537	0.767	0.813	0.896
		3	0.753	0.752	0.640	0.852	0.673	0.869
		**Average**	**0.742**	**0.761**	**0.615**	**0.825**	**0.747**	**0.877**
		95% CI	[0.686, 0.798]	[0.733, 0.790]	[0.504, 0.726]	[0.745, 0.905]	[0.634, 0.860]	[0.851, 0.903]

ACC, accuracy; SEN, sensitivity; SPE, specificity; PPV, positive predictive value; NPV, negative predictive value; CI, confidence interval; Bold Value, average value of 3 folds.

### Multimodal Prediction Models

**Figure 4:**
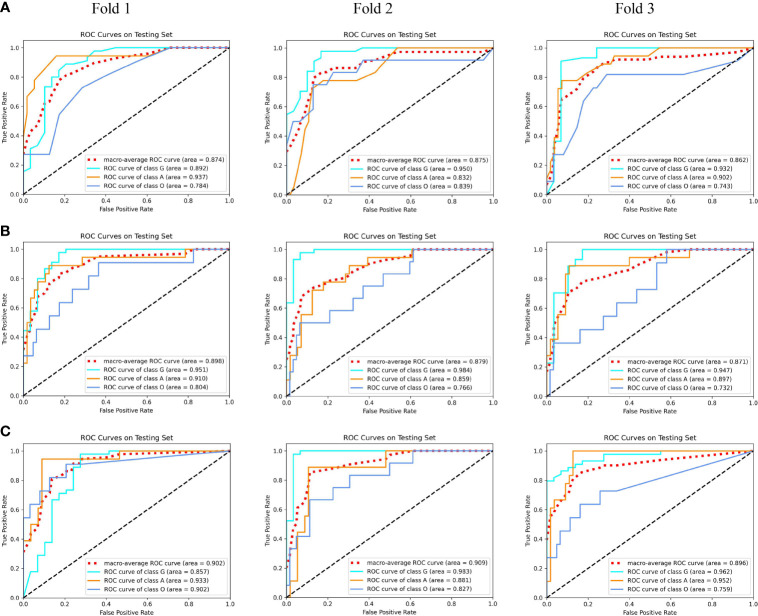
The receiver operating characteristic (ROC) curves of multimodal prediction models on three validation folds in our study. **(A)** Radiomics model. **(B)** Data fusion model. **(C)** Decision fusion model.

Using our proposed MMIDFNet method, through repeatedly adjusting the weights of each unimodal prediction model in inferring stage, we obtained the best multimodal prediction accuracy. After tuning the parameters of our radiomics model iteratively, we also obtained the best prediction using the fused features of multimodal images. In this paper, the multimodal prediction methods obtained with radiomics and MMIDFNet were named as radiomics model and decision fusion model, respectively. Specifically, considering our designed network also supports multi-channel input in the training stage, through inputting four modalities into four-channel input of our MMIDFNet simultaneously, we trained and obtained another multimodal prediction model (named as data fusion model) based on data fusion strategy for comparing the predictive performance between data fusion strategy and decision fusion strategy in our MMIDFNet method. Here, the data fusion strategy means that the multiple modal images were concatenated as input. The ROC curves of the radiomics model, data fusion model, and decision fusion model using multimodal MRI images on each validation fold are illustrated in **Figure 4**. We found that for the glioma subtypes “G”, and “A”, the prediction performance of all the methods was not greatly influenced by the splitting of the training and validation sets. However, for the glioma subtype “O”, the data splitting strategy had significant effects on the predicted AUC. In addition, the overall prediction of our decision fusion model for a multi-class predictive task is more balanced than the other two multimodal models.

**Figure 5:**
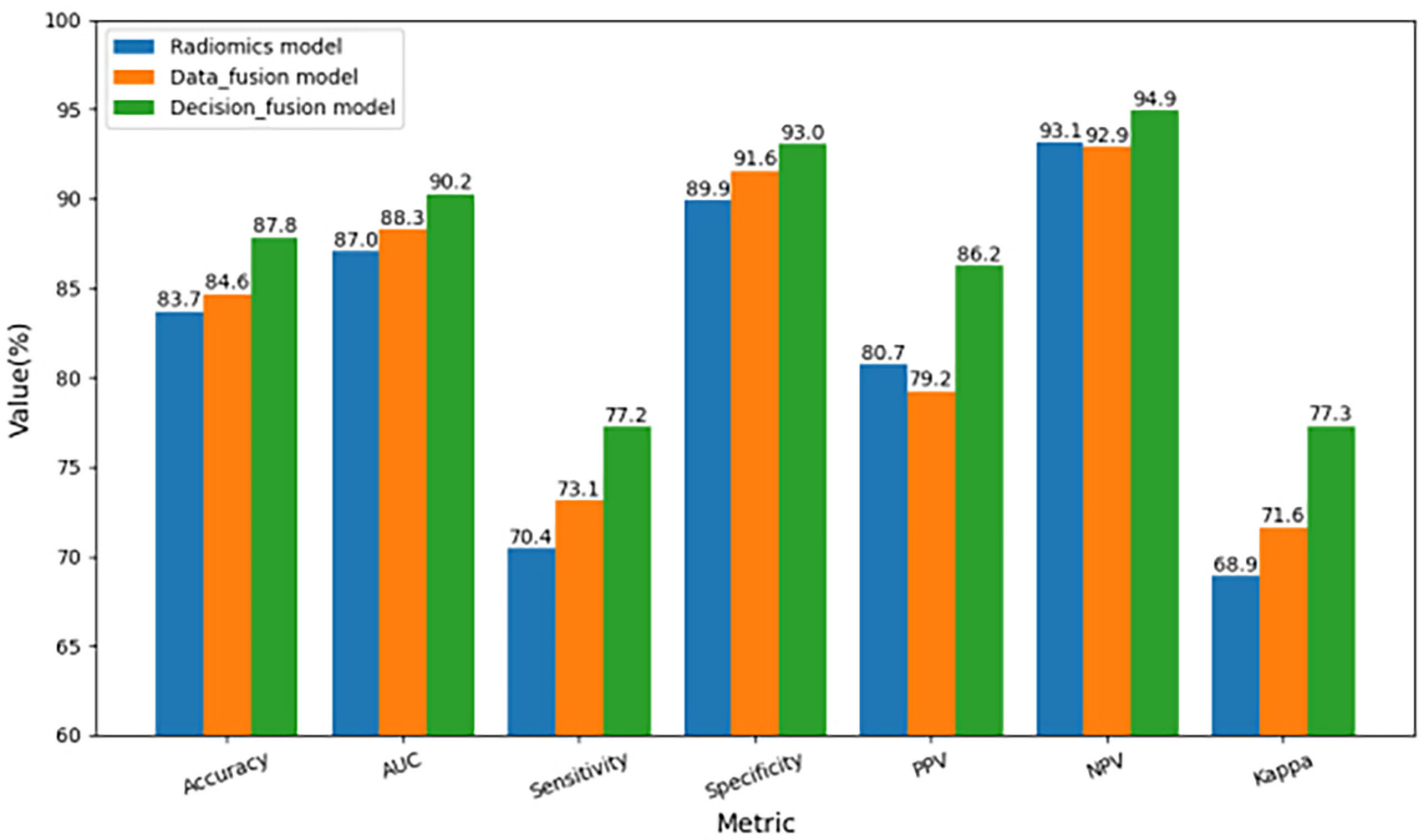
Comparison of three-fold cross-validation performance of the three multimodal prediction models.

To further compare the prediction performance of different fusion strategies, **Table 2** summarizes the 3-fold cross-validation performance of each method on the multimodal dataset. We found that the overall performance of our proposed fusion method was much better than radiomics and data fusion strategy, with the averaged accuracy increased by 4.9% and 3.8%, averaged AUC increased by 3.7% and 2.2%, averaged sensitivity increased by 9.7% and 5.6%, averaged specificity increased by 3.4% and 1.5%, averaged PPV increased by 6.8% and 8.8%, averaged NPV increased by 1.9% and 2.2%, averaged Kappa increased by 12.2% and 8.0%, respectively. According to **Table 2**, the averaged evaluation indices were also demonstrated with bar plots in **Figure 5** for better illustration.

**Table 2:** Three-fold cross-validation performance of multimodal prediction models using radiomics, data fusion strategy, and our proposed MMIDFNet methods.

Models	Fold	ACC	AUC	SEN	SPE	PPV	NPV	Kappa
Radiomics	1	0.851	0.874	0.702	0.885	0.905	0.945	0.699
	2	0.824	0.875	0.705	0.897	0.793	0.922	0.672
	3	0.836	0.862	0.706	0.914	0.724	0.927	0.695
	**Average**	**0.837**	**0.870**	**0.704**	**0.899**	**0.807**	**0.931**	**0.689**
	95% CI	[0.815,0.859]	[0.859,0.882]	[0.701, 0.708]	[0.875,0.922]	[0.661,0.954]	[0.912,0.951]	[0.665,0.712]
Data fusion	1	0.865	0.898	0.732	0.913	0.890	0.943	0.740
	2	0.838	0.879	0.744	0.926	0.741	0.922	0.713
	3	0.836	0.871	0.717	0.908	0.745	0.921	0.695
	**Average**	**0.846**	**0.883**	**0.731**	**0.916**	**0.792**	**0.929**	**0.716**
	95% CI	[0.820,0.872]	[0.860,0.905]	[0.709, 0.753]	[0.901,0.931]	[0.656,0.928]	[0.909,0.949]	[0.680,0.752]
Decision fusion	1	0.892	0.902	0.781	0.919	0.924	0.959	0.789
	2	0.865	0.909	0.741	0.926	0.821	0.949	0.749
	3	0.877	0.896	0.795	0.946	0.842	0.939	0.780
	**Average**	**0.878**	**0.902**	**0.772**	**0.930**	**0.862**	**0.949**	**0.773**
	95% CI	[0.856,0.900]	[0.892,0.913]	[0.727, 0.817]	[0.908,0.953]	[0.775,0.949]	[0.933,0.965]	[0.739,0.806]

ACC, accuracy; SEN, sensitivity; SPE, specificity; PPV, positive predictive value; NPV, negative predictive value; CI, confidence interval; Bold Value, average value of 3 folds.

Comparing **Table 2** with **Table 1**, we observed that the overall performance of multimodal models was superior to that of any model trained with unimodal MRI images, whether for the radiomics or our MMIDFNet method. Meanwhile, through using the multimodal model with multimodal MRI images, the difference between sensitivity and specificity was significantly reduced.

To further evaluate the effectiveness of our proposed method, we compared our method with state-of-the-art methods. The results are provided in **Table 3**. We observe that our decision fusion model achieves the highest averaged F1_score (0.878), Balanced_Acc (0.772), and Kappa (0.773) compared with the other methods. The F1_score of our method exceeds the methods of Pei at al (4)., Xue et al. (24), Pei et al. (25), and Yin et al. (26) at 5.9% (0.878 vs. 0.829), 13.9% (0.878 vs. 0.771), 13.9% (0.878 vs. 0.771), and 2.5% (0.878 vs. 0.857), respectively.

**Table 3:** Performance comparison of other state-of-the-art studies with ours.

Metrics	Pei et al. (4)	Xue et al. (24)	Pei et al. (25)	Yin et al. (26)	Radiomics	Data fusion	Decision fusion
F1_score 95% CI	0.829	0.771	0.771	0.857	0.837 [0.815, 0.859]	0.846 [0.820, 0.872]	**0.878** [0.856, 0.900]
Balanced_Acc 95% CI	0.749	NA	0.698	0.820	0.704 [0.701, 0.708]	0.731 [0.709, 0.753]	**0.772** [0.727, 0.817]
Kappa 95% CI	0.715	NA	0.627	0.767	0.689 [0.665, 0.712]	0.716 [0.680, 0.752]	**0.773** [0.739, 0.806]

CI, confidence interval; NA, not available; Bold Value, best value of the metric.

## Discussion

To further improve the performance of glioma subtype classification using MRI images only, we proposed a multimodal MRI image decision fusion-based network for glioma classification. In our method, during the training stage, we used the images of each MRI modality to train the network to obtain its best accuracy and obtained four pre-trained unimodal models. During the inferring stage, considering that different unimodal models have different predictive performance for three glioma subtypes, we assigned the weights for each unimodal model according to their classification capabilities to fully exploit their complementary predictive information of multi-class classification. Based on the decision fusion strategy, we assembled the four unimodal models together by using a linear weighted module and formed our multimodal prediction model for glioma subtype classification. The final predicted probabilities of the multimodal model were obtained by calculating the linear weighted sum of the predicted probabilities of the four pre-trained unimodal models. Thus, we improved the overall prediction performance of our multimodal prediction model by integrating the local predictive decision of each unimodal prediction model.

A finding of this study is that the decision fusion model using our MMIDFNet method outperformed the radiomics model based on radiomics in predicting glioma subtypes with multimodal MRI images (accuracy: 0.878 vs. 0.837). This is consistent with the findings of recent studies (38, 39). Moreover, as for unimodal models, our proposed MMIDFNet method also generated overall better results than our radiomics method. As described above, the radiomics-based methods comprised three tightly coupled stages: feature extraction, feature selection, and classification model building. Any small variations in each of the stages may affect the final prediction accuracy (39). Furthermore, compared to radiomics-based methods, deep learning-based methods are more flexible and superior in feature extraction since the hierarchy of features can be learned automatically from low level to high level in a layer-by-layer manner in the training phase (40).

It should be noted that the performance variability of brain tumor classification based on deep learning methods depends on the designed network architecture and trained hyper-parameters (41). Through the comparisons, we found that our MMIDFNet method performed better than the other four recent state-of-the-art methods based on the deep learning method (4, 24–26). This is mainly due to the adopted tumor segmentation algorithm, classification network, and image fusion strategy.

The region of a tumor lesion may have different image contrast properties in different imaging modalities (42). In contrast to other MRI modality images, the tumor boundary in the T1ce sequence is more significantly different from normal tissue, which facilitates automatic tumor segmentation. Besides, the T1ce sequence can better provide the condition of intratumoral so as to distinguish tumors from non-neoplastic lesions. Note that in our experimental results, either with the radiomics method or with our MMIDFNet method, the classification performance using T1ce modality images was significantly better than that using the other three modalities. These results are consistent with previous observations (25) and indicate that T1ce modality images should not be neglected in studies of glioma classification using multimodal MRI.

As for the glioma classification with multimodal MRI, how to mine rich feature representations across multimodal MRI images is the key factor in improving classification performance. Recent studies showed that image fusion can be operated at three levels: data, feature, and decision (27). Actually, as for our three multimodal prediction models, the radiomics model adopted the strategy of feature-level fusion, the data fusion model used the strategy of data-level fusion, and the decision fusion model employed the strategy of decision-level fusion. Our comparison results showed that, whatever the level of fusion, the accuracy of our multimodal models outperforms any models trained using unimodal MRI images, which indicates that each MRI modality can provide complementary features. In our radiomics method, through concatenating the four unimodal MRI features together, the accuracy of the RF classification model was raised to 0.837. Besides, as for our MMIDFNet method, the accuracy of the data fusion model (0.846) is 3.2% lower than that of the decision fusion model (0.878) *via* 3-fold cross-validation. This is mainly caused by the limitations of the data-level fusion strategy that does not fully take advantage of the features underlying each modality data and does not deal with how to fuse the features from the multimodal MRI images (33, 43, 44). However, in our decision fusion model, we used the weighting manner to ensemble the unimodal models in inferring stage. Theoretically, the fraction of each modality should be positively related to its contribution. From **Table 1**, we noticed that T1ce was the most useful modality for the prediction, followed by Flair, T2, and T1. In our fusion model, the weights for T1ce, Flair, T2, and T1 modalities are 2, 1, 0.7, and 0.3, respectively, which conform to the theoretical analysis and validate that our decision fusion model can fully explore the complementary information of different imaging modalities.

Brain tumor segmentation in MRI is of crucial importance for the subsequent diagnosis of brain tumors (45). Our efficient MMIDFNet method as well as radiomics method for glioma classification however relies on tumor segmentation performance. Any small variations in this stage may affect the final prediction performance and stability of the final prediction models. What cannot be ignored is that we employed a pre-trained tumor segmentation model from the BraTS challenge for tumor segmentation, while we did not use the ground truth delineated by experienced radiologists to segment the tumor regions from original MRI images. Although the segmentation result is not as accurate as the ground truth, we minimized the adverse effects caused by inaccurate segmentation by adopting the central clipping manner to segment out the whole tumor regions.

Although encouraging, our method has several limitations. First, as a retrospective study, the sample size of the dataset used in the present study was limited, which has an adverse effect on the robustness of our designed model. Therefore, the few-shot learning method may be a better choice to handle the problem. Second, we used only MRI modalities in the present study without considering other types of data, especially pathological whole-slide images. To further improve the performance of glioma subtype classification, in the future, we could try to combine MRI, pathology images, molecular genetic information, and other clinical data to conduct a multi-omics clinical study. Third, the number of subtype “G” in our training set was about 60.2%, and this resulted in the class imbalance issue. To handle this issue, we used the focal loss function through balancing the loss of different subtypes, which indeed alleviated the issue. However, this scheme might not be optimal because it ignores the differences in data distribution. Therefore, to deal with this common problem in medical images classification, more effective measures for forming more balanced data will be considered in our future work.

In conclusion, we studied the preoperative glioma subtype classification by developing a multimodal MRI image decision fusion-based network based on a deep learning technique. Through designing a linear weighted module to assemble the unimodal models trained with unimodal MRI images together, our multimodal prediction model fully mined the complementary information of multimodal MRI images. Extensive experimental results showed that the proposed MMIDFNet method was superior to recent state-of-the-art methods, which suggests its potential use in clinical practice for glioma subtype classification based on only MRI images.

## Data Availability Statement

The original contributions presented in the study are included in the article/supplementary material. Further inquiries can be directed to the corresponding authors.

## Author Contributions

SG, LHW, and YZ: conceptualization, methodology, software, visualization, and writing. QC and LW: collection and assembly of data. SG, JZ, and LW: data analysis and interpretation. All authors: writing and final approval of the manuscript.

## Funding

This work was supported in part by the National Natural Science Foundation of China under Grant 62161004, in part by the Natural Science Foundation of Guizhou Province under Grant Qiankehe J No. [2020]1Y255, in part by the Program PHC-Cai Yuanpei 2018 under Grant 41400TC, in part by the Guizhou Province Education Department Project under Grant Qianjiaohe KY[2016]321, and in part by the Guizhou Science and Technology Plan Project under Grant Qiankehe ZK[2021]002.

## Conflict of Interest

The authors declare that the research was conducted in the absence of any commercial or financial relationships that could be construed as a potential conflict of interest.

## Publisher’s Note

All claims expressed in this article are solely those of the authors and do not necessarily represent those of their affiliated organizations, or those of the publisher, the editors and the reviewers. Any product that may be evaluated in this article, or claim that may be made by its manufacturer, is not guaranteed or endorsed by the publisher.

## Footnotes

1
https://miccai.westus2.cloudapp.azure.com/competitions/1

2
https://github.com/Project-MONAI/MONAI

3
https://pyradiomics.readthedocs.io/en/latest/index.html
